# Group mindfulness-based cognitive therapy associated with reduced professional caregiver burden and improved quality of care in dementia: a multi-method study

**DOI:** 10.3389/fneur.2026.1880966

**Published:** 2026-08-24

**Authors:** Longyun Chen, Bencheng Wang, Ting Li, Nan Li, Xuanxuan Chen, Fei Liu, Tao Lv

**Affiliations:** 1 Shanghai Changning Mental Health Center, Affiliated Mental Health Center of East China Normal University, Shanghai, China; 2 Shanghai Yangsi Hospital, Shanghai, China; 3 The Affiliated Mental Health Center of Kunming Medical University, Yunnan Mental Hospital, Kunming, Yunnan, China; 4 The People’s Hospital of Deyang, Deyang, Sichuan, China

**Keywords:** caregiver burden, mindfulness-based cognitive therapy, multi-method study, neuropsychiatric symptoms, professional dementia caregivers

## Abstract

**Purpose:**

To evaluate the association between an 8-week group Mindfulness-Based Cognitive Therapy (MBCT) program and changes in professional dementia caregiver burden, perceived stress, and patient neuropsychiatric symptoms.

**Methods:**

This retrospective multi-method study included 94 professional dementia caregivers at a psychiatric hospital in China (intervention: *n* = 48; control: *n* = 46). The intervention group participated in an 8-week group MBCT program, while the control group received routine education. Caregiver burden (Neuropsychiatric Inventory Caregiver Distress Scale [NPI-D]), perceived stress (Perceived Stress Scale-10 [PSS-10]), and patient symptoms (Brief Psychiatric Rating Scale [BPRS], Behavioral Pathology in Alzheimer’s Disease Rating Scale [BEHAVE-AD], Neuropsychiatric Inventory [NPI]) were assessed at baseline and five follow-up points over 20 weeks. Semi-structured interviews with 18 intervention participants explored perceived intervention mechanisms. Quantitative data were analyzed using repeated measures ANOVA; qualitative data were analyzed using reflexive thematic analysis.

**Results:**

The intervention group demonstrated significantly greater reductions in caregiver burden (NPI-D: from 32.15 ± 8.53 to 19.90 ± 5.71) and perceived stress (PSS-10: from 23.83 ± 4.23 to 16.82 ± 3.94) compared to the control group from week 8 through week 20 (all *p* < 0.001). Significant group × time interactions were observed for BPRS (*F* = 2.682, *p* = 0.021), BEHAVE-AD (*F* = 2.747, *p* = 0.018), and NPI severity (*F* = 2.353, *p* = 0.040), with patient improvements emerging at week 12, independent of antipsychotic medication adjustments. Correlation analysis revealed positive associations between caregiver burden reduction and patient symptom improvement (*r* = 0.36–0.44, all *p* < 0.001). Qualitative findings identified five themes: development of mindful awareness, cognitive reappraisal of patient behaviors, cultivation of self-compassion, value of group support, and barriers and facilitators to sustained practice.

**Conclusion:**

The 8-week group MBCT program was associated with sustained reductions in caregiver burden and perceived stress, accompanied by improvements in patient neuropsychiatric symptoms among professional dementia caregivers. The group format provided additional perceived benefits through peer support and experience normalization. These findings support further investigation of group-based mindfulness interventions within professional dementia care settings using randomized controlled designs.

## 1 Introduction

Dementia represents a growing global health challenge, with an estimated 55 million people affected worldwide and projections indicating this number will reach 152 million by 2050 (1). Professional caregivers in institutional settings bear a substantial portion of the daily care demands, often managing complex neuropsychiatric symptoms (NPS) including agitation, aggression, and disinhibition under conditions of high workload, shift rotations, and limited psychological support (2, 3). Research indicates that up to 78% of dementia caregivers experience significant burden (4, 5), and professional caregivers face unique occupational stressors, such as emotional labor, compassion fatigue, and institutional pressures, that differ from those encountered by family caregivers (6).

Neuropsychiatric symptoms in persons with dementia emerge as the strongest predictors of caregiver stress and psychological distress (7). The cumulative effect of caregiving stress not only undermines caregiver well-being but may also compromise care quality and patient outcomes (8). This bidirectional relationship between caregiver mental health and quality of care provision underscores the critical need for effective psychosocial interventions targeting caregiver well-being.

Mindfulness-Based Cognitive Therapy (MBCT), which integrates mindfulness meditation with cognitive-behavioral techniques, has demonstrated efficacy in reducing psychological distress across various populations (9, 10). Modified MBCT programs for dementia caregivers have shown significant reductions in perceived stress, with effects sustained at 3- to 6-month follow-up (11). The group format offers additional therapeutic value through shared experience normalization, mutual emotional support, and collective problem-solving, which may be particularly beneficial for professional caregivers who experience occupational isolation (12).

Despite promising evidence, most existing MBCT studies have focused on family caregivers and caregiver-centered outcomes, with limited examination of effects on patient behavioral symptoms. Additionally, the mechanisms underlying MBCT’s effectiveness in professional caregiving contexts remain insufficiently understood, and evidence from Chinese cultural settings is limited (13). This multi-method study therefore evaluated the association between an 8-week group MBCT program and changes in professional caregiver burden and patient neuropsychiatric symptoms at a psychiatric hospital in China, complemented by qualitative exploration of perceived intervention mechanisms.

## 2 Methods

### 2.1 Study design

This retrospective multi-method study analyzed data from professional dementia caregivers who participated in a mindfulness-based intervention program at a secondary-grade A psychiatric hospital in China between March 2023 and December 2023. The study employed a non-randomized controlled design with two components: (1) a quantitative comparison of an 8-week group MBCT intervention (*n* = 48) versus routine care education (*n* = 46), and (2) a qualitative exploration of intervention experiences through semi-structured interviews with a subset of intervention participants (*n* = 18). Following a multi-method design (14), the two components were conducted and analyzed independently, with complementary findings discussed in the discussion section.

This study followed the Transparent Reporting of Evaluations with Nonrandomized Designs (TREND) statement for the quantitative component (15) and the Consolidated Criteria for Reporting Qualitative Research (COREQ) guidelines for the qualitative component (16).

The study protocol was approved by the Ethics Committee (approval number: M202142). All participants provided written informed consent at the time of program enrollment, and additional ethics approval was obtained for secondary data use and post-intervention qualitative interviews.

### 2.2 Participants

Caregivers were eligible if they met the following criteria: (a) formal hospital employees or contracted care workers serving as primary caregivers for DSM-5 diagnosed dementia patients; (b) aged 18 years or older; (c) employed as caregivers (minimum 40 h per week) for at least 6 months in the dementia care unit; and (d) able to communicate in Mandarin Chinese. Exclusion criteria included: (a) current participation in other psychological interventions; (b) severe psychiatric disorders requiring hospitalization; and (c) incomplete baseline or follow-up assessment data. Care recipients were required to have a confirmed dementia diagnosis with a Neuropsychiatric Inventory (NPI) total score ≥ 4, indicating the presence of clinically relevant neuropsychiatric symptoms (17).

Caregivers in the intervention group had voluntarily enrolled in the MBCT program based on interest and scheduling availability. Control group caregivers were identified from the same clinical setting during the same period among caregivers who did not enroll in the MBCT program and who received routine dementia care education. Whether individual control caregivers had expressed interest in participating in MBCT was not systematically recorded. Therefore, group allocation reflected voluntary enrollment and scheduling availability rather than random assignment.

### 2.3 Intervention

The intervention group participated in an 8-week group MBCT program adapted for professional dementia caregivers. The program was based on the standard MBCT protocol (18), with modifications informed by mindfulness-based interventions adapted for dementia caregivers (19) to address the specific challenges of professional dementia care. Groups consisted of 8–12 caregivers facilitated by two certified MBCT therapists with specialized training in dementia care. Sessions were held weekly for 2 h at the hospital.

The program integrated formal mindfulness practices (body scan, sitting meditation, mindful movement, 3-min breathing space) with cognitive therapy techniques targeting caregiving-related automatic thoughts and emotional reactions. Specific adaptations included: contextualized examples applying mindfulness to common challenging behaviors (repetitive questioning, nighttime wandering, and resistance to care); exercises cultivating non-judgmental acceptance of patient symptoms; discussion of compassion fatigue and self-compassion; strategies for integrating brief mindfulness into caregiving routines; and role-playing scenarios of mindful responding to high-stress situations. To support workplace application, facilitators provided scenario-based guidance on using brief mindfulness practices during routine care and acute stress situations, including bathing assistance, aggressive or agitated behavior, repeated requests, nighttime wandering, and shift transitions. Participants practiced these applications through role-play, group troubleshooting of recent work situations, and discussion of how to adapt the 3-min breathing space when leaving the care area was not feasible. The detailed session-by-session curriculum is provided in Supplementary Table S1. Session attendance was recorded by the facilitators. Participants were provided with audio recordings and encouraged to practice for 20–30 min daily. Engagement with the audio recordings was encouraged but was not objectively monitored, and participants did not keep formal practice logs. An informal peer support messaging group was maintained between sessions.

The control group received routine dementia care education consisting of monthly educational sessions on dementia pathophysiology, symptom management, and safety considerations, without mindfulness training, cognitive restructuring, or group emotional support components. Control caregivers did not receive MBCT during the 20-week follow-up period, and all study outcome assessments were completed before any subsequent mindfulness-based training opportunities. After completion of the evaluation period, the hospital continued to offer MBCT-based staff training opportunities to interested staff, including caregivers who had previously been in the control group; participation in any subsequent training was not part of the present analysis.

### 2.4 Outcome measures

Caregiver burden was assessed using the Neuropsychiatric Inventory Caregiver Distress Scale (NPI-D) (20), which evaluates distress associated with 12 neuropsychiatric symptom domains on a 6-point scale (0 = not distressing at all to 5 = extremely distressing; total range: 0–60). Perceived stress was measured using the Perceived Stress Scale-10 (PSS-10) (21), a 10-item instrument rated on a 5-point Likert scale (0 = never to 4 = very often; total range: 0–40).

Secondary outcomes included: the Brief Psychiatric Rating Scale (BPRS) (22), an 18-item clinician-rated instrument (total range: 18–126); the Behavioral Pathology in Alzheimer’s Disease Rating Scale (BEHAVE-AD) (23), a 25-item caregiver-based instrument (total range: 0–75), and the NPI Patient Severity Score (24), assessing 12 neuropsychiatric domains (total range: 0–144). Antipsychotic medication use was recorded as chlorpromazine equivalent dosage (mg/day).

### 2.5 Data collection

Quantitative assessments were conducted at six time points: baseline (week 0), week 4, week 8 (post-intervention), week 12, week 16, and week 20. All assessments were administered by trained research assistants blinded to group allocation. For this retrospective analysis, two independent researchers extracted data from medical records, with discrepancies resolved through verification against source documents.

Qualitative data were collected between March and May 2024, approximately 3–12 months after intervention completion. All 48 intervention completers were invited to participate in semi-structured interviews; 18 completed interviews. Purposive sampling ensured representation across response levels: high responders (≥50% NPI-D reduction, *n* = 7), moderate responders (25–49% reduction, *n* = 6), and minimal responders (<25% reduction, *n* = 5). Interviews lasted 45–70 min (mean = 56 min) and followed a semi-structured guide covering five domains: (1) perceived changes in stress management; (2) specific mindfulness practices and effectiveness; (3) role of the group format; (4) barriers to sustained practice; and (5) perceived changes in caregiving approaches. Interviews were conducted by two trained interviewers (one clinical psychologist and one qualitative researcher) who had not been involved in delivering the MBCT intervention, and were audio-recorded and transcribed verbatim. The principal investigator was involved in the design and oversight of the original MBCT program but did not deliver the intervention or conduct interviews. The interviewers had no prior relationship with participants, minimizing social desirability bias. Reflexive journals were maintained throughout the analysis process to document analytical decisions and potential biases.

### 2.6 Statistical analysis

Quantitative analyses were performed using SPSS version 26.0. Baseline comparability was assessed using independent samples t-tests for continuous variables and chi-square tests for categorical variables. Between-group differences at each time point were examined using independent samples *t*-tests, and within-group changes were evaluated using paired *t*-tests. Repeated measures ANOVA was conducted to examine group × time interactions. Pearson correlation coefficients were calculated to examine associations between changes in caregiver burden and patient symptoms. All analyses used a two-tailed *α* = 0.05.

Qualitative transcripts were analyzed using reflexive thematic analysis following Braun and Clarke (25). NVivo 12 was used to facilitate data organization, coding, retrieval, and comparison across transcripts. Two researchers independently read the transcripts, generated initial codes inductively, compared recurring codes across participants, grouped related codes into candidate themes, and refined theme names and boundaries through consensus discussions. Trustworthiness was enhanced through investigator triangulation (independent dual coding with 87.3% initial agreement, reaching 95.6% after consensus discussions), member checking (summary findings shared with five participants for verification), an audit trail documenting analytical decisions, and thick description of participant context and experiences. Data saturation was assessed prospectively during data collection and achieved after 15 interviews, with three additional interviews confirming identified themes.

## 3 Results

### 3.1 Participant characteristics

Baseline demographic and clinical characteristics are presented in Table 1. The two groups were comparable on all baseline variables including caregiver age, gender, education level, marital status, patient age, patient gender, dementia type, and disease duration (all *p* > 0.05).

**Table 1:** Baseline demographic and clinical characteristics.

Characteristic	Intervention group (*n* = 48)	Control group (*n* = 46)	Test statistic	*p*-value
Caregiver characteristics				
Age, years	53.96 ± 4.13	53.39 ± 5.13	0.595	0.554
Gender			0.180	0.672
Male	10 (20.83)	8 (17.39)		
Female	38 (79.17)	38 (82.61)		
Education, years	7.13 ± 2.18	7.83 ± 2.45	1.465	0.146
Marital status			0.548	0.459
Divorced	4 (8.33)	6 (13.04)		
Married, *n* (%)	44 (91.67)	40 (86.96)		
Patient characteristics				
Age, years	76.04 ± 5.81	74.76 ± 6.34	1.021	0.310
Sex			0.180	0.672
Male	10 (20.83)	8 (17.39)		
Female	38 (79.17)	38 (82.61)		
Dementia type			0.148	0.929
Alzheimer’s disease	32 (66.67)	30 (65.22)		
Vascular dementia	8 (16.67)	7 (15.22)		
Other	8(16.67)	9(19.57)		
Disease duration, years	5.69 ± 2.31	5.93 ± 2.34	0.502	0.617

### 3.2 Primary outcomes

**Figure 1:**
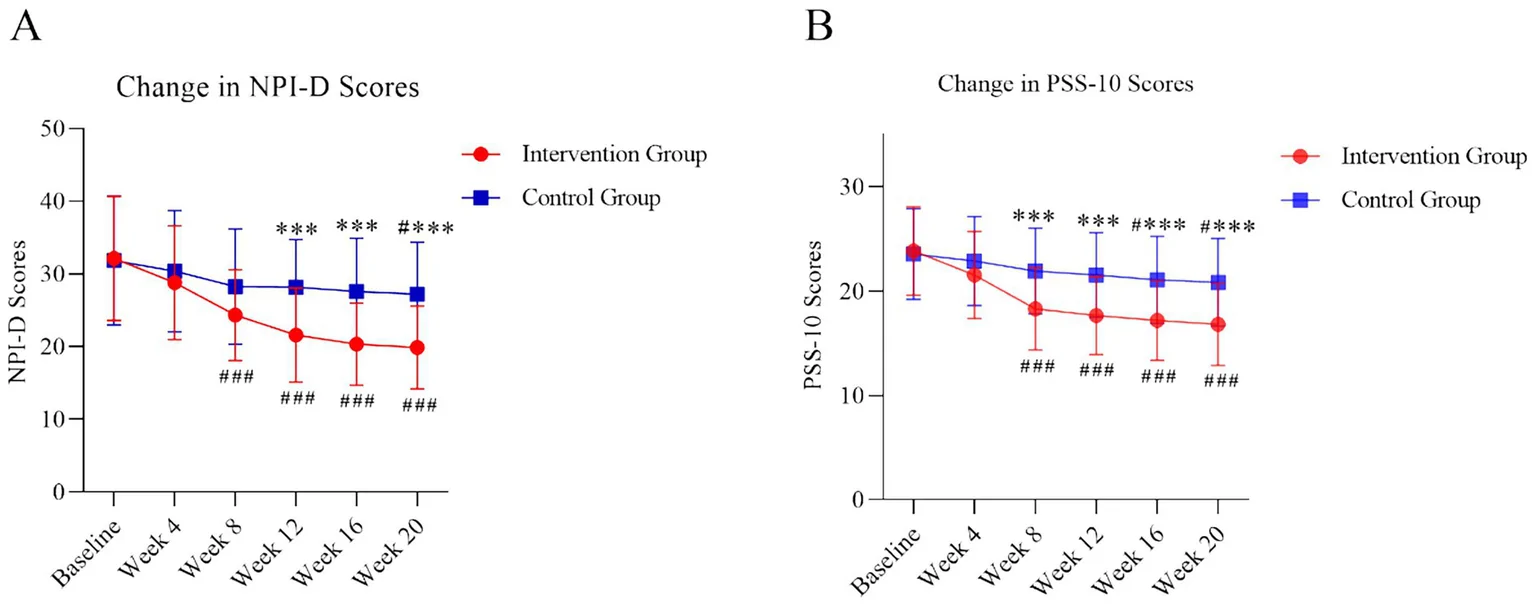
Primary outcomes: changes in caregiver burden and perceived stress over time. Trajectories of **(A)** NPI-D scores and **(B)** PSS-10 scores across six assessment time points (baseline, weeks 4, 8, 12, 16, and 20). Data are mean ± SD. *^#^p* < 0.05, *^##^p* < 0.01, *^###^p* < 0.001 vs. baseline; *p < 0.05, **p < 0.01, ***p < 0.001 for between-group comparison.

NPI-D scores over 20 weeks are shown in Figure 1A. Groups were comparable at baseline (intervention: 32.15 ± 8.53; control: 31.85 ± 8.89). The intervention group showed significant reductions from week 8 through week 20 compared to baseline (week 8: 24.35 ± 6.27; week 12: 21.60 ± 6.48; week 16: 20.35 ± 5.65; week 20: 19.90 ± 5.71; all *p* < 0.001), while the control group remained stable throughout (week 20: 27.23 ± 7.18). Between-group differences were significant from week 12 through week 20 (all *p* < 0.001). Repeated measures ANOVA revealed significant main effects for time (*F* = 20.240, *p* < 0.001), group (*F* = 51.230, *p* < 0.001), and time × group interaction (*F* = 4.563, *p* < 0.001).

PSS-10 scores are presented in Figure 1B. Baseline scores were comparable between groups (intervention: 23.83 ± 4.23; control: 23.54 ± 4.36). The intervention group demonstrated significant reductions from week 8 through week 20 compared to baseline (week 8: 18.30 ± 3.93; week 12: 17.67 ± 3.75; week 16: 17.19 ± 3.81; week 20: 16.82 ± 3.94; all *p* < 0.001), while the control group showed minimal changes (week 20: 20.85 ± 4.22). Between-group differences were significant from week 8 through week 20 (all *p* < 0.001). Repeated measures ANOVA revealed significant main effects for time (*F* = 20.900, *p* < 0.001), group (*F* = 63.280, *p* < 0.001), and time × group interaction (*F* = 4.548, *p* < 0.001).

### 3.3 Secondary outcomes

**Figure 2:**
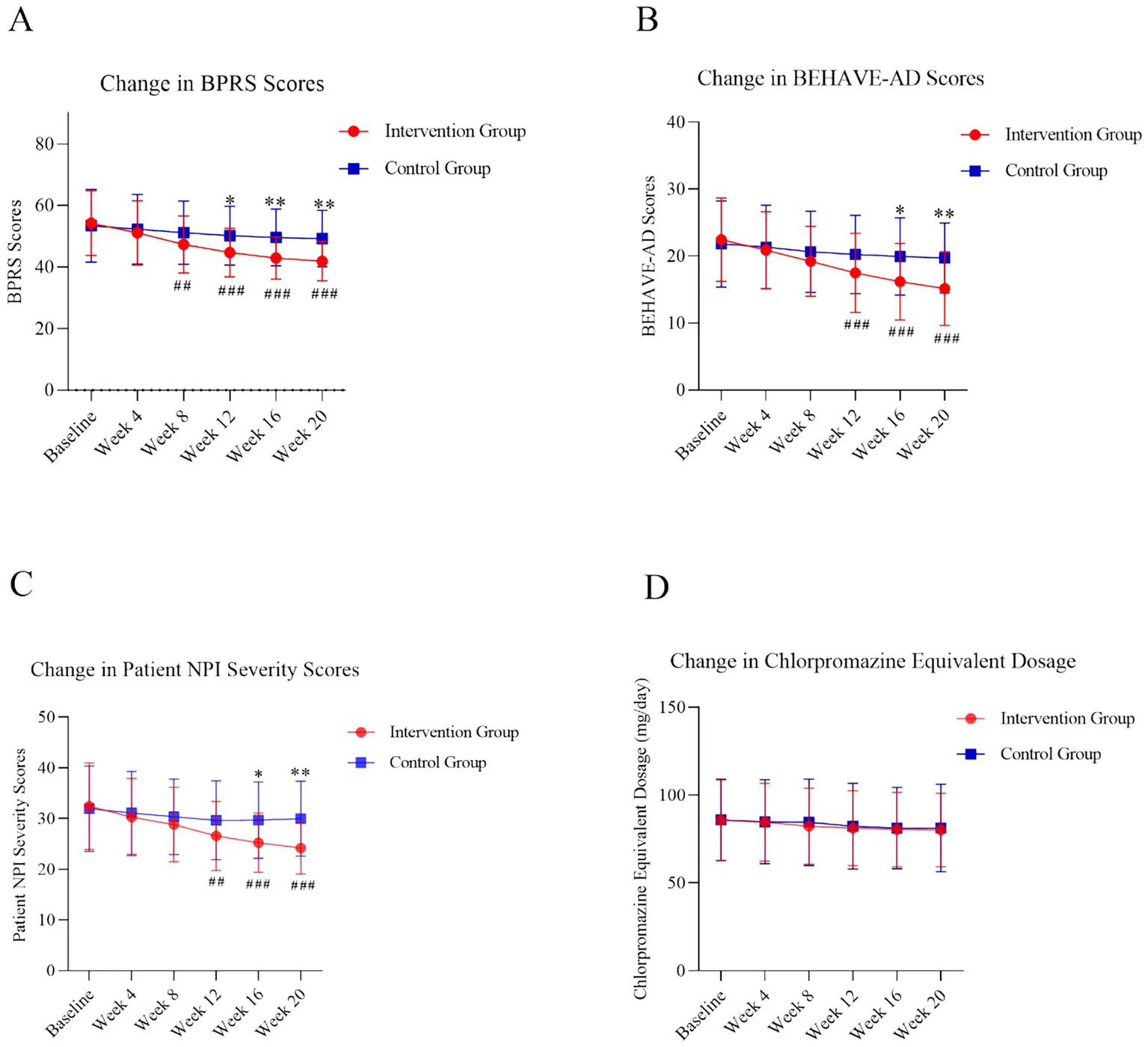
Secondary outcomes: changes in patient neuropsychiatric symptoms and medication use over time. Trajectories of **(A)** BPRS scores, **(B)** BEHAVE-AD scores, **(C)** NPI patient severity scores, and **(D)** chlorpromazine equivalent dosages across six assessment time points (baseline, weeks 4, 8, 12, 16, and 20). Data are mean ± SD. *^#^p* < 0.05, *^##^p* < 0.01, *^###^p* < 0.001 vs. baseline; *p < 0.05, **p < 0.01, ***p < 0.001 for between-group comparison.

Patient symptom measures and chlorpromazine equivalent dosages are shown in Figure 2. The intervention group demonstrated significant reductions in BPRS scores from week 8 onward (all *p* < 0.01 vs. baseline), with between-group differences emerging at week 12 (*p* < 0.05) and persisting at weeks 16 and 20 (both p < 0.01). BEHAVE-AD scores in the intervention group were significantly lower from week 12 onward (all *p* < 0.01 vs. baseline), with between-group differences at weeks 16 and 20 (*p* < 0.05 and *p* < 0.01, respectively). NPI patient severity scores followed a similar pattern, with significant within-group reductions from week 12 and between-group differences at weeks 16 and 20.

Repeated measures ANOVA showed significant group × time interactions for BPRS (*F* = 2.682, *p* = 0.021), BEHAVE-AD (*F* = 2.747, *p* = 0.018), and NPI severity (*F* = 2.353, *p* = 0.040). Chlorpromazine equivalent dosages did not differ significantly between groups at any time point, and no significant time, group, or interaction effects were observed (all *p* > 0.05), indicating that changes in caregiver burden and patient symptoms were independent of medication adjustments.

### 3.4 Association between caregiver burden and patient symptoms

Correlation analyses (Table 2) revealed significant positive associations between reductions in NPI-D scores and reductions in patient BPRS scores (*r* = 0.41, *p* < 0.001), BEHAVE-AD scores (*r* = 0.36, *p* < 0.001), and NPI patient severity scores (*r* = 0.44, *p* < 0.001), suggesting that improvements in caregiver psychological functioning were associated with improvements in patient behavioral and psychiatric symptoms.

**Table 2:** Correlations between changes in caregiver burden and patient symptoms (baseline to week 20).

Variables	Pearson *r*	95% CI	*P*-value
ΔNPI-D vs. ΔBPRS	0.41	0.230–0.568	<0.001
ΔNPI-D vs. ΔBEHAVE-AD	0.36	0.171–0.521	<0.001
*Δ*NPI-D vs. ΔNPI-patient	0.44	0.259–0.590	<0.001

Δ indicates change from baseline to week 20. NPI-D, Neuropsychiatric Inventory Caregiver Distress Scale; BPRS, Brief Psychiatric Rating Scale; BEHAVE-AD, Behavioral Pathology in Alzheimer’s Disease Rating Scale; NPI, Neuropsychiatric Inventory; CI, confidence interval.

### 3.5 Qualitative findings

Using the inductive thematic analysis process described above, five interrelated themes were identified from 18 semi-structured interviews. Themes were generated from recurring code clusters across transcripts, including caregivers’ descriptions of noticing automatic reactions, reinterpreting patient behaviors, reducing self-criticism, receiving peer support, and negotiating barriers to practice in routine care. Representative quotations were selected by consensus to illustrate the central meaning of each theme and are presented in Table 3. The interview sample consisted of 14 females and 4 males, aged 43–58 years (mean = 52.83 ± 4.75), with education levels ranging from 6 to 12 years (mean = 7.83 ± 2.03). Non-participation was primarily due to work schedule constraints (*n* = 12), shift conflicts (*n* = 8), and lack of interest (*n* = 5). Among the 26 who initially agreed, 8 were unable to complete interviews due to schedule changes (*n* = 5) and personal reasons (*n* = 3). Themes 1–3 represent individual-level perceived mechanisms of change, while Themes 4–5 address contextual factors. Data saturation was achieved after 15 interviews, with three additional interviews confirming identified themes.

**Table 3:** Representative qualitative quotes by theme.

Theme	Subtheme	Representative quote	Participant
Theme 1: Developing Mindful Awareness of Automatic Reactions	Recognizing stress signals	“Before the program, when a patient asked me the same question repeatedly during my shift, I would feel my frustration building. Now I can notice that rising tension in my chest, that tightness in my shoulders. I pause, take three breaths like we practiced, and then I can respond more professionally.”	P7, female, age 58
	Applying the 3-min breathing space	“I keep a small card with the breathing space instructions in my uniform pocket. When things get intense during my shift, I excuse myself briefly—maybe step into the staff room for 3 min—do the breathing exercise, and return with a clearer mind.”	P4, female, age 46
Theme 2: Cognitive Reappraisal of Patient Behaviors	Reframing disease vs. person	“The turning point for me was understanding at a deeper level that ‘the disease is doing this, not the person.’ I knew this intellectually from my training, but the mindfulness practice helped me embody this understanding.”	P15, male, age 51
	Reducing personalization	“I used to interpret patients’ constant demands and call button use as them being ‘difficult’ or questioning my care competence. Through the group discussions, I realized I was personalizing behaviors that are actually symptoms.”	P2, female, age 49
Theme 3: Cultivation of Self-Compassion and Acceptance	Reduced self-criticism	“I used to be very hard on myself—thinking I should be more patient, I should handle every situation perfectly, I should never feel frustrated. The self-compassion exercises in the program were transformative.”	P9, female, age 56
	Accepting disease progression	“The hardest but most helpful lesson was accepting that I cannot stop the disease progression. I cannot restore their memories or reverse the decline, no matter how excellent my care is.”	P6, female, age 58
Theme 4: Value of Group Support and Shared Experience	Normalization of struggles	“I thought I was the only one feeling angry and overwhelmed by this work. I felt like I was failing as a professional caregiver. But in the first group session, when another colleague shared that she sometimes fantasizes about transferring to a different unit, I felt enormous relief.”	P14, female, age 56
	Sustained peer support	“We created a WeChat group that’s still active. When I’m having a particularly difficult shift, I can message the group and someone always responds with understanding or a practical suggestion.”	P13, female, age 58
Theme 5: Barriers and Facilitators to Sustained Practice	Facilitator: integrating into care tasks	“I could not consistently find 30 min outside of work to sit and meditate, but I discovered I could practice mindfulness during routine care tasks at work. When bathing a patient, I focus on the warmth of the water, the gentle movements, staying present.”	P16, female, age 58
	Minimal responder perspective	“I attended all the sessions and tried to participate, but I never quite understood how to apply these concepts during my actual work shifts. ‘Observing thoughts without judgment’—what does that mean when a patient is hitting you?”	P17, female, age 54
	High responder perspective	“Mindfulness has fundamentally changed my relationship with this demanding work. It’s not that the job is easier—patients are still declining, behaviors are still challenging—but my experience of the work is completely different.”	P1, female, age 51

#### 3.5.1 Theme 1: developing mindful awareness of automatic reactions

Caregivers described increased recognition of their automatic emotional responses to challenging patient behaviors. Participants reported that techniques such as the 3-min breathing space enabled them to create a deliberate pause between stimulus and response during stressful work situations, facilitating more intentional professional responses. This theme was generated from repeated accounts of noticing bodily stress signals, emotional escalation, and the use of a deliberate pause before responding to patients.

#### 3.5.2 Theme 2: cognitive reappraisal of patient behaviors

Participants reported shifts in interpreting patient symptoms, moving from viewing challenging behaviors as personally directed to understanding them as neurological disease manifestations. This reframing was particularly emphasized by high responders as central to reducing their occupational stress and feelings of professional inadequacy. This theme was generated from repeated descriptions of shifting from personalized interpretations of patient behavior toward dementia-related explanations.

#### 3.5.3 Theme 3: cultivation of self-compassion and acceptance

Caregivers described reduced self-criticism regarding their professional performance and increased acceptance of personal limitations. Several participants noted that self-compassion paradoxically enhanced their capacity for patient care, as reduced self-blame freed emotional resources for compassionate engagement with patients. This theme reflected recurring accounts of reduced self-blame, greater acceptance of professional limitations, and preservation of emotional resources for patient care.

#### 3.5.4 Theme 4: value of group support and shared experience

The group format was consistently identified as a critical component. Participants emphasized the normalization of their struggles through hearing colleagues’ experiences, which reduced feelings of professional isolation. Collective problem-solving was particularly valued, with caregivers sharing practical strategies for managing specific challenging behaviors. Several participants maintained informal peer support networks through a messaging application after program completion. This theme was generated from accounts emphasizing normalization, shared problem-solving, and continued peer support beyond formal sessions.

#### 3.5.5 Theme 5: barriers and facilitators to sustained practice

Work schedule constraints and physical exhaustion were identified as primary barriers to maintaining formal daily meditation practice. However, many participants successfully integrated brief informal mindfulness into routine care tasks (e.g., mindful presence during patient bathing, mindful breathing during shift transitions). Key facilitators included perceiving tangible workplace benefits early in the program, supervisor support, and institutional provision of quiet spaces for practice. Minimal responders more frequently reported difficulty applying abstract mindfulness concepts to concrete workplace situations and lower engagement with practice assignments. This theme was generated from contrasting accounts of successful integration of brief mindfulness into care routines versus difficulty translating abstract mindfulness concepts into high-pressure work situations.

The correspondence between quantitative outcomes and qualitative themes is presented in Table 4. The matrix illustrates how reductions in caregiver burden corresponded with cognitive reappraisal of patient behaviors (Theme 2), how perceived stress reduction related to both mindful awareness (Theme 1) and self-compassion (Theme 3), and how sustained effects were supported by peer networks (Theme 4) and integration of practice into daily routines (Theme 5).

**Table 4:** Complementary findings matrix: correspondence between quantitative outcomes and qualitative themes.

Quantitative finding	Complementary qualitative theme	Interpretation
Significant reduction in NPI-D scores from week 8 through week 20 (*p* < 0.001)	Theme 2: Cognitive Reappraisal – Caregivers reframed patient behaviors as disease manifestations rather than personal provocations	Cognitive reframing of challenging behaviors may represent a key psychological mechanism underlying the observed reduction in caregiver burden
Significant reduction in PSS-10 scores from week 8 through week 20 (*p* < 0.001)	Theme 1: Mindful Awareness — creating a deliberate “pause” between stimulus and response; Theme 3: Self-Compassion — reduced self-criticism and guilt regarding professional performance	Interruption of automatic reactive patterns (Theme 1) and release of emotional resources through self-compassion (Theme 3) may jointly contribute to reduced perceived stress through complementary pathways
Patient symptom improvements (BPRS, NPI) emerged later (week 12) than caregiver improvements (week 8)	Themes 1 and 2: Caregivers described first changing their own response patterns, then observing gradual changes in patient interactions	The temporal lag supports a proposed pathway in which caregiver behavioral change precedes and contributes to patient symptom improvement
Differential response levels observed across participants	Theme 5: Minimal responders reported difficulty applying abstract mindfulness concepts to concrete workplace situations and lower practice engagement	Individual differences in intervention response may be linked to the ability to translate abstract concepts into practical workplace application
Intervention effects maintained at week 20 (12 weeks post-intervention)	Theme 4: Sustained peer support via messaging group; Theme 5: Integration of informal mindfulness into routine care tasks	Peer support networks and adaptation of practice to workplace routines may serve as mechanisms for maintaining intervention effects beyond the formal program

## 4 Discussion

This multi-method study suggests that group MBCT may be a promising approach for supporting professional dementia caregivers in institutional dementia care. The qualitative themes offer a possible explanation for the observed patterns, as participants described mindfulness practice as a practical way to pause during stressful care interactions, reinterpret patient behaviors as dementia-related symptoms, reduce self-blame, and sustain coping through peer support. These interpretations remain preliminary given the retrospective, non-randomized design, but they help situate the observed associations within the everyday realities of professional dementia care.

The sustained reductions in caregiver burden observed in the intervention group are consistent with systematic review findings suggesting medium to large effect sizes for mindfulness-based interventions on caregiver outcomes, with effects persisting beyond the intervention period (9). The MBCT program was specifically adapted to address professional caregiving challenges, such as responding mindfully to repetitive questioning and resistance to care, which likely enhanced its relevance and practical utility for this population. This adaptation approach is consistent with Stone et al. (26) and aligns with evidence supporting modified MBCT for dementia caregivers (11). The maintenance of observed changes through week 20 may partly reflect participants’ reported use of informal workplace mindfulness practices in daily routines, as described in the qualitative interviews. These findings are particularly relevant given that professional dementia caregivers face unique occupational stressors, including emotional labor, compassion fatigue, and institutional pressures (6, 27), which distinguish their support needs from those of family caregivers. The observed reductions in perceived stress and caregiver burden suggest that group MBCT may help address these occupational demands by equipping caregivers with skills to manage chronic workplace stress, a factor closely related to burnout risk.

The concurrent reductions in caregiver burden and patient symptom measures provide correlational support for the bidirectional relationship between caregiver psychological functioning and care recipient behavioral disturbances (4, 8, 28). The observed correlations between reductions in caregiver burden and improvements in patient symptom measures suggest that caregiver well-being and patient outcomes changed in parallel. However, given the non-randomized design, these associations should be interpreted cautiously and cannot establish directionality. The temporal pattern, with caregiver benefits emerging at week 8 and patient improvements at week 12, suggests that patient benefits may accrue gradually as caregivers consistently apply mindfulness-informed approaches over time. The mechanisms underlying patient symptom improvements likely include reduced caregiver reactivity to challenging behaviors, creation of a calmer care environment, and improved emotional regulation that minimizes conflict escalation. Notably, these changes occurred independently of antipsychotic medication adjustments, supporting the potential of caregiver-focused interventions as a non-pharmacological strategy for addressing neuropsychiatric symptoms (29).

The qualitative themes, generated from recurring code clusters rather than predetermined categories, provide complementary insights into perceived intervention mechanisms. Together, the themes suggested a practical pathway by which participants perceived change, in which mindful awareness helped caregivers notice automatic stress reactions and pause before responding, cognitive reappraisal supported reinterpretation of agitation, repetitive questioning, and resistance to care as dementia-related symptoms rather than personal provocations, and self-compassion reduced self-blame when care situations remained difficult. These themes align with theoretical models proposing that caregiver appraisals mediate the relationship between neuropsychiatric symptoms and burden (30, 31), and with evidence that self-kindness may protect against burnout in healthcare contexts (32). Theme 5 also helped contextualize variation in response, as minimal responders more often described difficulty translating abstract mindfulness concepts into high-pressure care situations.

The group format emerged as a valued intervention component, with participants emphasizing normalization, collective problem-solving, and sustained peer support networks. These findings are consistent with evidence that group-based formats facilitate a sense of community and shared identity (33–35). The therapeutic value of universality, recognizing that others face similar challenges, may be particularly beneficial for professional caregivers who experience occupational isolation. Many participants maintained informal peer support through a messaging application after program completion, suggesting that workplace-based mindfulness programs may catalyze long-term collegial support systems extending beyond the formal intervention period. For professional dementia caregivers, the group format appears to offer therapeutic value beyond mindfulness skills training alone.

Several limitations should be considered. The non-randomized, retrospective design limits causal inference. Although baseline comparability was demonstrated, selection bias remains possible, as caregivers who volunteered for MBCT may have been more motivated or open to psychological interventions. In particular, because interest in MBCT among control caregivers was not systematically documented, unmeasured differences in motivation or openness to psychological interventions may have contributed to selection bias. The absence of an attention-control condition precludes attribution of effects to mindfulness-specific mechanisms versus nonspecific factors such as therapist attention or group support. The modest sample size (*N* = 94) and single-site recruitment limit generalizability, particularly given potential cultural factors influencing mindfulness acceptability in Chinese populations. The qualitative interviews were conducted 3–12 months post-intervention, which may affect recall accuracy. However, this delay was necessitated by the retrospective study design and may have facilitated reflection on sustained behavioral changes rather than immediate post-intervention reactions. The qualitative participation rate (37.5% of intervention completers) raises questions about response bias, although purposive sampling across response levels partially addresses this concern. Additionally, formal and informal mindfulness practice, including engagement with the audio recordings, was not systematically monitored, preventing dose–response analysis. Future studies should incorporate practice logs to quantify practice engagement among professional caregivers.

These preliminary findings suggest that group MBCT programs adapted for professional dementia caregivers may be feasible and acceptable in Chinese institutional care settings. Nurses and care managers may consider incorporating brief mindfulness techniques (e.g., the 3-min breathing space) into professional development programs for dementia care staff. Institutional support, including designated quiet spaces and supervisor endorsement, was identified as a key facilitator of sustained practice and may enhance program implementation. Future randomized controlled trials with active control conditions and longer-term follow-up are warranted to confirm these associative findings and determine optimal intervention parameters. Additionally, comparative effectiveness research is needed to determine whether group MBCT offers advantages over other psychosocial interventions for professional dementia caregivers, and cost-effectiveness analyses would inform resource allocation decisions within healthcare systems.

## 5 Conclusion

This multi-method study found that an 8-week group MBCT program adapted for professional Chinese dementia caregivers was associated with reductions in caregiver burden and perceived stress, accompanied by improvements in patient neuropsychiatric symptoms. The group format was perceived to provide additional benefits through peer support and experience normalization. Qualitative findings suggest that mindful awareness, cognitive reappraisal of patient behaviors, and self-compassion may serve as potential mechanisms underlying these associations. These findings contribute to the evidence base supporting mindfulness-based interventions for professional dementia caregivers and warrant confirmation through randomized controlled trials with active control conditions.

## Data Availability

The original contributions presented in the study are included in the article/Supplementary material, further inquiries can be directed to the corresponding authors.
