# Supplementary material for: Group mindfulness-based cognitive therapy associated with reduced professional caregiver burden and improved quality of care in dementia: a multi-method study
Source: Front Neurol. 2026 Aug 24;17:1880966. doi: 10.3389/fneur.2026.1880966 (PMC13547055; doi:10.3389/fneur.2026.1880966)
Supplement: Supplementary file 1 [file Table_1.docx]

**Supplementary Table S1. Session-by-Session MBCT Curriculum for Professional Dementia Caregivers**

| **Session** | **Theme** | **Core Mindfulness Practice** | **Caregiving-Specific Adaptation** | **Home Practice Assignment** |
| --- | --- | --- | --- | --- |
| 1 | Automatic Pilot | Mindful eating; Body scan | Recognizing automatic reactions to patient behaviors (e.g., repetitive questioning) | Body scan (20 min daily) |
| 2 | Dealing with Barriers | Body scan; Thoughts and feelings exercise | "Being" vs. "doing" mode during caregiving tasks | Body scan alternating with sitting meditation |
| 3 | Mindfulness of Breath and Body | Sitting meditation (breath focus) | Using breath as anchor during stressful caregiving moments | Sitting meditation (20 min daily); 3-minute breathing space |
| 4 | Staying Present with Difficulty | Sitting meditation (expanding awareness) | Recognizing aversion patterns with challenging patient behaviors | Sitting meditation; Mindful walking during shifts |
| 5 | Acceptance and Letting Be | Sitting meditation (acceptance) | Distinguishing changeable vs. unchangeable aspects of dementia care | Sitting meditation with acceptance focus |
| 6 | Thoughts Are Not Facts | Sitting meditation (thoughts as mental events) | Identifying catastrophic interpretations of caregiving situations | Noting automatic thoughts during care interactions |
| 7 | Self-Care and Burnout Prevention | Mindful movement (gentle yoga) | Identifying personal warning signs of stress escalation; Setting professional boundaries | Activity scheduling; Pleasant and unpleasant events calendar |
| 8 | Consolidation and Relapse Prevention | Review of all practices | Developing individualized plan for maintaining mindfulness in caregiving routines | Personalized practice plan (formal + informal) |

*Note: All sessions were 2 hours in duration and included: guided mindfulness exercises (30–40 min), didactic presentation (15–20 min), group discussion (40–50 min), and practice review with assignment (10–15 min).*
