# Supplementary material for: Group mindfulness-based cognitive therapy associated with reduced professional caregiver burden and improved quality of care in dementia: a multi-method study
Source: Front Neurol. 2026 Aug 24;17:1880966. doi: 10.3389/fneur.2026.1880966 (PMC13547055; doi:10.3389/fneur.2026.1880966)
Supplement: Supplementary file 2 [file Table_2.docx]

**Supplementary File 2. COREQ Checklist**

*Consolidated Criteria for Reporting Qualitative Research (COREQ): 32-Item Checklist for Interviews and Focus Groups*

**Domain 1: Research Team and Reflexivity**

| **Item No.** | **Guide Question** | **Response / Location in Manuscript** |
| --- | --- | --- |
| 1 | Interviewer/facilitator: Which author(s) conducted the interviews? | Two trained interviewers: one clinical psychologist and one qualitative researcher (Section 2.5) |
| 2 | Credentials: What were the researcher’s credentials? | Clinical psychologist (PhD) and qualitative researcher (PhD) (Section 2.5) |
| 3 | Occupation: What was their occupation at the time of the study? | University researchers affiliated with the hospital (Section 2.5) |
| 4 | Gender: Was the researcher male or female? | One female, one male |
| 5 | Experience and training: What experience or training did the researcher have? | Both trained in qualitative interviewing; neither involved in MBCT delivery (Section 2.5) |
| 6 | Relationship established: Was a relationship established prior to study? | No prior relationship with participants (Section 2.5) |
| 7 | Participant knowledge: What did participants know about the researcher? | Informed that interviewers were researchers exploring MBCT program experiences |
| 8 | Interviewer characteristics: What characteristics were reported? | Positionality statement provided in Section 2.5 |

**Domain 2: Study Design**

| **Item No.** | **Guide Question** | **Response / Location in Manuscript** |
| --- | --- | --- |
| 9 | Methodological orientation: What methodological orientation was stated? | Reflexive thematic analysis following Braun and Clarke (2006) (Section 2.6) |
| 10 | Sampling: How were participants selected? | Purposive sampling across response levels (high, moderate, minimal) (Section 2.5) |
| 11 | Method of approach: How were participants approached? | In person at workplace (Section 2.5) |
| 12 | Sample size: How many participants were in the study? | n = 18 (Section 2.5) |
| 13 | Non-participation: How many people refused or dropped out? | 30 of 48 did not participate; reasons in Section 3.5 |
| 14 | Setting: Where was the data collected? | Private conference room at the hospital (Section 2.5) |
| 15 | Presence of non-participants: Was anyone else present? | No |
| 16 | Description of sample: Important characteristics of the sample? | Demographics and response levels in Section 3.5 |
| 17 | Interview guide: Were questions/prompts/guides provided? | Yes; five-domain semi-structured guide described in Section 2.5 |
| 18 | Repeat interviews: Were repeat interviews carried out? | No |
| 19 | Audio/visual recording: Did the research use recording? | Audio-recorded with participant permission (Section 2.5) |
| 20 | Field notes: Were field notes made? | Reflexive journals maintained (Section 2.5) |
| 21 | Duration: What was the duration of the interviews? | 45–70 minutes (mean = 56 min) (Section 2.5) |
| 22 | Data saturation: Was data saturation discussed? | Yes; achieved after 15 interviews, confirmed by 3 additional (Section 3.5) |
| 23 | Transcripts returned: Were transcripts returned for comment? | Transcripts checked by interviewers; summary findings shared with 5 participants for member checking (Section 2.6) |

**Domain 3: Analysis and Findings**

| **Item No.** | **Guide Question** | **Response / Location in Manuscript** |
| --- | --- | --- |
| 24 | Number of data coders: How many coders coded the data? | Two independent coders (Section 2.6) |
| 25 | Coding tree: Did authors describe the coding tree? | Five major themes with subthemes in Section 3.5 and Table 3 |
| 26 | Derivation of themes: Identified in advance or derived from data? | Inductively derived from data (Section 2.6) |
| 27 | Software: What software was used? | NVivo 12 (QSR International) (Section 2.6) |
| 28 | Participant checking: Did participants provide feedback? | Yes; member checking with 5 participants (Section 2.6) |
| 29 | Quotations: Were quotations presented? | Yes; representative quotes in Table 3 |
| 30 | Data and findings consistent? | Yes; quotes directly support theme descriptions |
| 31 | Clarity of major themes? | Five themes clearly delineated (Section 3.5) |
| 32 | Clarity of minor themes: Diverse cases or discussion? | Differential experiences by response level described (Theme 5; Table 3) |
